# Worldwide Variation in Human Milk Metabolome: Indicators of Breast Physiology and Maternal Lifestyle?

**DOI:** 10.3390/nu10091151

**Published:** 2018-08-23

**Authors:** Melvin C. L. Gay, Petya T. Koleva, Carolyn M. Slupsky, Elloise du Toit, Merete Eggesbo, Christine C. Johnson, Ganesa Wegienka, Naoki Shimojo, Dianne E. Campbell, Susan L. Prescott, Daniel Munblit, Donna T. Geddes, Anita L. Kozyrskyj, InVIVO LactoActive Study Investigators

**Affiliations:** 1 School of Molecular Sciences, University of Western Australia, Perth, WA 6009, Australia; melvin.gay@uwa.edu.au (M.C.L.G.); donna.geddes@uwa.edu.au (D.T.G.); 2 Department of Pediatrics, University of Alberta, Edmonton, Alberta, AB T6G 1C9, Canada; koleva@ualberta.ca; 3 Departments of Nutrition/Food Science & Technology, University of California Davis, California, CA 95616-5270, USA; cslupsky@ucdavis.edu; 4 Division of Medical Microbiology, University of Cape Town, Cape Town, Rondebosch 7701, South Africa; elloisedutoit@gmail.com; 5 Department of Environmental Exposure and Epidemiology, Norwegian Institute of Public Health, Oslo N-0213, Norway; merete.eggesbo@fhi.no; 6 Department of Public Health Sciences, Henry Ford Hospital, Detroit, MI 48208, USA; cjohnso1@hfhs.org (C.C.J.); gwegien1@hfhs.org (G.W.); 7 Department of Pediatrics, Chiba University, Chiba 260-8677, Japan; shimojo@faculty.chiba-u.jp; 8 Department of Allergy and Immunology, Children’s Hospital at Westmead, University of Sydney, Sydney, NSW 2145, Australia; dianne.campbell1@health.nsw.gov.au; 9 School of Medicine, University of Western Australia, Nedlands, WA 6009, Australia; Susan.Prescott@telethonkids.org.au; 10 ORIGINS Project, Telethon Kids Institute, Perth Children’s Hospital, Perth, WA 6009, Australia; 11 Department of Paediatrics, Imperial College London, London W2 1NY, UK; daniel.munblit08@imperial.ac.uk; 12 Faculty of Pediatrics, Sechenov University, Moscow 119991, Russia; 13 Departments of Pediatrics/Obstetrics & Gynecology, School of Public Health, University of Alberta, Edmonton, Alberta AB T6G 1C9, Canada

**Keywords:** human milk, milk metabolites, lactation, milk metabolomics

## Abstract

Human milk provides essential substrates for the optimal growth and development of a breastfed infant. Besides providing nutrients to the infant, human milk also contains metabolites which form an intricate system between maternal lifestyle, such as the mother’s diet and the gut microbiome, and infant outcomes. This study investigates the variation of these human milk metabolites from five different countries. Human milk samples (*n* = 109) were collected one month postpartum from Australia, Japan, the USA, Norway, and South Africa and were analyzed by nuclear magnetic resonance. The partial least squares discriminant analysis (PLS-DA) showed separation between either maternal countries of origin or ethnicities. Variation between countries in concentration of metabolites, such as 2-oxoglutarate, creatine, and glutamine, in human milk, between countries, could provide insights into problems, such as mastitis and/or impaired functions of the mammary glands. Several important markers of milk production, such as lactose, betaine, creatine, glutamate, and glutamine, showed good correlation between each metabolite. This work highlights the importance of milk metabolites with respect to maternal lifestyle and the environment, and also provides the framework for future breastfeeding and microbiome studies in a global context.

## 1. Introduction

Human milk provides all of the essential nutrients and bioactive substrates required for optimal growth and development of the nursing infant [1]. This includes not only large immunoglobulins and proteins, but also numerous low molecular weight substances, such as simple sugars and complex human milk oligosaccharides (HMOs), amino acids, short-chain fatty acids, and other energy metabolic pathway intermediates [2]. These nutrients work as part of a complex functional unit, operating in concert with intestinal enzymes to influence infant physiology. Many metabolites, such as HMOs, lactose, and other milk sugars, are also fermented by gut microbiota to generate additional metabolites. Variation in the human milk metabolome, namely with HMOs, is seen by maternal phenotype and diet [3]. Despite this, there are still only a few studies reporting on the determinants of small molecule concentrations in human milk or their role, and only limited studies on global regional differences in other milk components [4,5].

Patterns of infant growth are strong predictors of future cognitive performance and cardiometabolic health [6,7,8]. Breastfeeding may protect from rapid growth during infancy and the risk for overweight/obesity, diabetes, and high blood pressure [9]; however, this may vary with compositional factors in human milk [10]. Because human milk metabolites influence infant gut microbial composition, and gut microbial dysbiosis has been linked to future overweight [11,12], geographic variations in small milk molecules can conceivably predict regional differences in infant gut microbial development, infant growth, and future health. The activity of some milk metabolites directly reflects maternal diet, and hence would be a function of regional differences in dietary intake [13]. Taking as an example immune system activation of spillover lactose in the colon secondary to lactase deficiency in the small intestine [14], the activity of still other milk metabolites may be subject to regional differences in host genetics. With an aim to learn more about these less well-studied small molecular weight metabolites, we undertook a descriptive study of human milk metabolomics, comparing women from diverse geographical locations. Healthy women without atopic conditions were the focus of the comparison. We found regional differences in milk metabolites related to lactation performance that differentiated South African women from women from other countries.

## 2. Materials and Methods 

With representation across ethnicity, maternal atopy status, and infant sex, 109 milk samples collected one month after birth were selected from six existing international cohorts: Perth, Australia (*n* = 29 from 2 cohorts which include 21 atopic mothers, i.e., Perth #1, [15] and 8 non-atopic mothers, i.e., Perth #2) [16]; Chiba, Japan (*n* = 12); Detroit, USA (*n* = 18) [17]; Oslo, Norway (*n* = 40) [18]; and Cape Town, South Africa (*n* = 10) [4]. The one-month postpartum time period was selected because human milk composition has stabilized by then [2]. Maternal atopy status was defined according to maternal report of asthma, eczema, allergies, or other atopic diseases, or at least one blood allergen-specific IgE level ≥ 35 kU/L for dust mite, dog, cat, Timothy grass, ragweed, *Alternaria alternate,* egg, or German cockroach. Research ethics approval was obtained from the Human Research Ethics Committee of The University of Western Australia, Human Research Ethics Committee of the Princess Margaret Hospital, Committee on Human Research of Chiba University, Institutional Review Board at Henry Ford Health System, Norwegian Regional Committees for Medical and Health Research Ethics, and University of Cape Town Human Research Ethical Committee.

Before sample collection, the mothers were given oral and written instructions for standardized collection of samples. Human milk samples were collected manually or with an electric breast pump into a sterile tube. Prior to collection, nipples and mammary areola were cleaned with soap and sterile water, and for the samples from South Africa, the area was additionally soaked with chlorhexidine to reduce contamination by skin microbes. All of the samples were kept frozen at −20 °C until delivery to the laboratory and then stored at −20 °C or −80 °C until further analysis. All the samples were shipped to Edmonton, Canada for storage, processing and Nuclear Magnetic Resonance (NMR) analysis at The Metabolomics Innovation Centre.

Milk metabolite levels were determined by NMR because of its high reproducibility and coverage of a large range of metabolites. Prior to NMR spectroscopy, milk samples were thawed on ice and mixed thoroughly. Approximately 500 μL of each sample was filtered to remove residual lipids and protein using Amicon Ultra 0.5 mL 3-kDa cutoff spin filter (Millipore Sigma, Burlington, MA, USA). The filtration was performed at 10,000× *g* for 15 min at 4 °C. Then, a 350 μL clear filtrate was placed in a 1.5-mL Eppendorf tube, followed by the addition of 70 μL of D_2_O and 60 μL of standard buffer solution (585 mM NaHPO_4_ (pH 7.0), 11.667 mM disodium-2,2-dimethyl-2-silapentane-5-sulfonate (DSS), and 0.47% NaN_3_ in H_2_O). The samples (460 μL) were then transferred to a regular NMR tube for subsequent NMR spectral analysis. All ^1^H-NMR spectra were collected on a Varian 500 MHz Inova spectrometer equipped with a 5-mm HCN Z-gradient pulsed-field gradient cryogenic probe. ^1^H-NMR spectra were acquired at 25 °C using the first transient of the Varian tnnoesy pulse sequence, which was chosen for its high degree of selective water suppression and quantitative accuracy of resonances around the solvent. Water suppression pulses were calibrated to achieve a bandwidth of 80 G. Spectra were collected with 128 transient and 8 steady-state scans using a 4-s acquisition time (48,000 complex points) and a 1-s recycle delay. Quality control (QC) mixtures which consisted of 4 metabolites at 1 mM were analyzed for every 20 to 25 samples, and a relative standard deviation of <2% was observed.

Before spectral analysis, all free induction decays were zero-filled to 64,000 data points and line broadened to 0.5 Hz. The methyl singlet produced by a known quantity of DSS was used as an internal standard for chemical shift referencing (set to 0 ppm) and for quantification. All ^1^H-NMR spectra were processed and analyzed using the Chenomx NMR Suite Professional software package version 8.1 (Chenomx Inc., Edmonton, AB, Canada). The Chenomx NMR Suite software allows for qualitative and quantitative analysis of an NMR spectrum by manually fitting spectral signatures from an internal database to the spectrum. Typically, 90% of visible peaks were assigned to a compound, and more than 90% of the spectral area could be routinely fit using the Chenomx spectral analysis software. Most of the visible peaks were annotated with a compound name and expressed as µmol/L.

### Statistical Analysis

Partial Least Square Discriminant Analysis (PLS-DA) was created using the Excel add-in Multibase 2015 package (Numerical Dynamics, Tokyo, Japan). PLS-DA was performed in order to maximize the separation between the different countries as well as the ethnicities of the mothers. Data preparation was made using the scaling method of standard deviations. Sample scatterplot and loading plots were compared where significant variables which contribute to sample distribution can be easily identified. Statistical analyses were carried out using R studio 1.1.414 (Rstudio Inc., Boston, MA, USA) with package nlme for linear mixed models to test for significant differences between the milk metabolites among different countries, and among ethnicities of the mothers. Milk metabolite levels were compared with each other using the Pearsons correlation across all the non-atopic mothers using Package corrplot [19]. Differences are considered to be significant if *p* < 0.05.

## 3. Results

Of the 109 participating women, 69% were Caucasian, 51% were nursing male infants, and 43% had a history of atopy; this comparison oversampled atopic women and their additional results are the subject of another paper. The majority of South African women were of mixed race and all were non-atopic; most of the comparator cohorts were of Caucasian ancestry. Of the women in the US cohort, 39% were African American (labelled as Black) and only one African-American woman was non-atopic. All women had delivered vaginally and did not receive intrapartum antibiotics.

### 3.1. Milk Metabolite Clusters by Country

**Figure 1:**
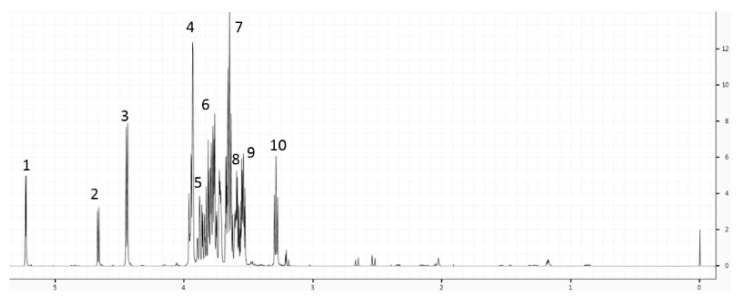
A representative ^1^H nuclear magnetic resonance (NMR) spectrum of human milk. The ^1^H chemical shifts for lactose are annotated 1 to 10.

A total of 28 metabolites were identified in the human milk of our descriptive study, including sugars (fucose, glucose, lactose), amino acids (alanine, glutamine, glutamate, glycine, isoleucine, leucine, valine), choline and its metabolites, and energy metabolites (acetone, citrate, creatine, creatine phosphate, creatinine, lactate, 2-oxoglutarate, pyruvate, succinate), as shown in Figure 1. Most of the measured metabolites were within the range of those reported in other studies at a comparable time postpartum [20,21].

**Figure 2:**
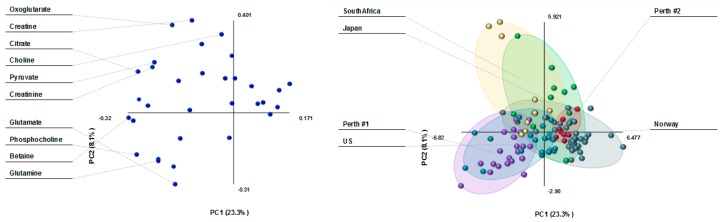
Partial Least Square Discriminant Analysis (PLS-DA) loading plot (**left**) and scatterplot (**right**) of human milk metabolites from 109 women in various countries. The score plot shows separation based on maternal country of origin (purple: Perth #1, atopic mothers; red: Perth #2, non-atopic mothers; green: Japan; aqua: US; dark green: Norway; and yellow: South Africa). The loading plot shows the milk metabolites that influence the separation based on maternal country of origin.

The PLS plot in Figure 2 shows three main clusters of milk metabolites in women by country as follows: (1) South African, (2) Australian atopic and US, and (3) Australian non-atopic and Norwegian cohorts. Milk metabolites of Japanese women overlapped between those of Norwegian and South African women. Creatine and 2-oxoglutarate were the main drivers of milk metabolite differences between South Africa and other countries. Glutamine and phosphocholine differentiated the milk of Norwegian and US women.

### 3.2. Milk Metabolite Differences in Healthy, Non-Atopic Women

Milk metabolite composition varied to the greatest extent between South African, and Norwegian or Australian women (Table 1). When compared to those of Norwegian women, the following milk metabolites were higher in concentration in South African women: lactose (*p* = 0.02), 2-oxoglutarate (*p* < 0.001), citrate (<0.001) and creatine (*p* ≤ 0.001)/creatine-phosphate (*p* = 0.02) /creatinine (*p* < 0.001), as well as betaine (*p* < 0.001) and glycerol-phosphocholine (*p* = 0.02). Except for 2-oxoglurate and glycerol-phosphocholine, similar differences in these milk metabolites were observed between South African and Australian women. No country differences were observed in other milk sugars (glucose or fucose) or other energy metabolite (i.e., succinate) concentrations.

In addition, a statistical significance was also noted for lower milk levels of glutamine (*p* < 0.001) in Norwegian versus US women. Japanese women had significantly higher milk levels of pyruvate (*p* < 0.01) and lactate (*p* < 0.01) than Norwegian women. Milk levels of methanol were significantly lower (*p* < 0.02) in Norway versus all other countries.

### 3.3. Milk Metabolite Correlations

**Figure 3:**
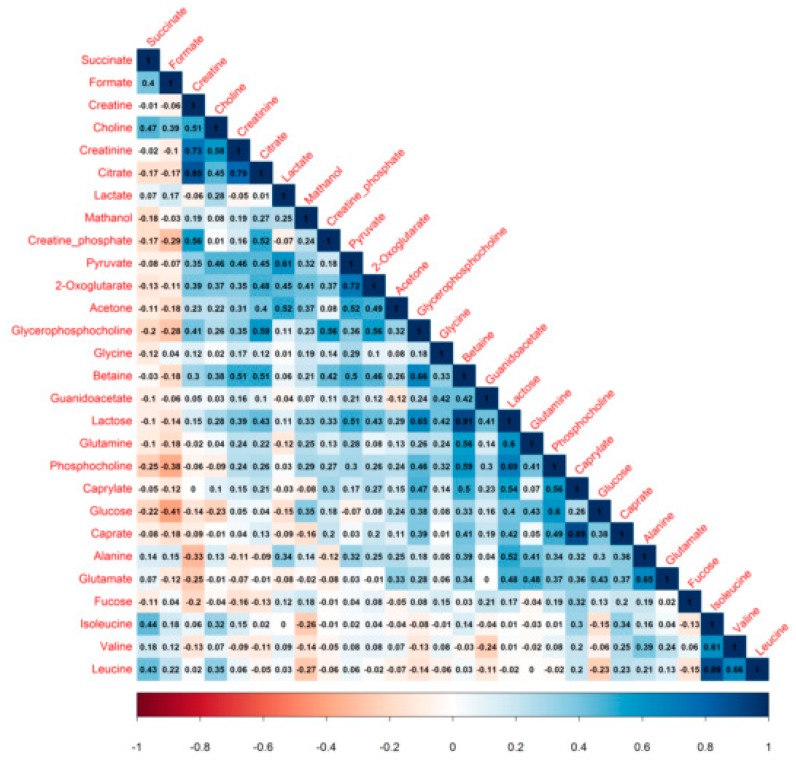
Correlation plot of 28 human milk metabolites from non-atopic mothers. Values shows Pearson correlation coefficients between pairs of metabolites. Positive correlation, zero correlation and negative correlation are represented by colors ranging from blue to white to red, respectively.

Correlations between milk metabolites are reported in Figure 3. Both lactose and betaine were positively correlated with citrate, creatine, and the phosphocholines; milk lactose levels significantly rose with those of 2-oxoglutarate.

### 3.4. Milk Metabolite Clusters by Race/Ethnicity

**Figure 4:**
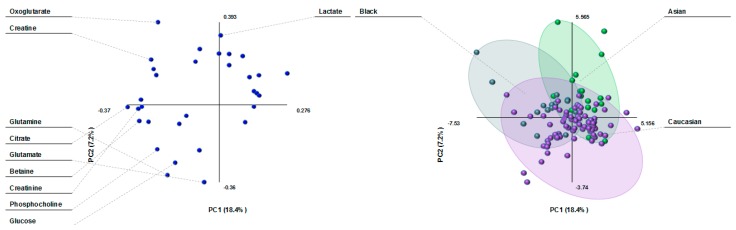
PLS-DA loading plot (**left**) and scatterplot (**right**) of human milk metabolites from women of different ethnic groups. The score plot shows separation based on maternal ethnicity (purple: Caucasian; green: Asian; and dark green: Black). The loading plot shows the milk metabolites that influence the separation based on maternal ethnicity.

Metabolite variation by ethnicity in the PLS plots was similar to country differences, showing three clusters of milk composition: Black, Caucasian and Asian (Figure 4). Milk lactate differentiated the Asian population from the rest. 2-Oxoglutarate and creatine were the main drivers of the milk cluster in the Black population, whereas the main milk metabolites in the Caucasian cluster were glutamate, glutamine, glucose, and phosphocholine.

### 3.5. Milk Metabolite Variation by Ethnicity in Healthy, Non-Atopic Women

2-Oxoglutarate (*p* = 0.008) and creatine (*p* = 0.02)/creatine phosphate (*p* = 0.008)/creatinine (*p* = 0.03), as well as betaine (*p* = 0.01) and glycerophosphocholine (*p* = 0.009) concentrations were higher in the milk of Black compared to Caucasian women (Table 2). Although lactose levels were also higher, this difference did not reach statistical significance (*p* = 0.07). In contrast, milk valine levels were lower in Black than in Caucasian women. In essence, these results compare mixed-race South African to Caucasian women, because only one US Black-race woman was a member of the Black race group. Compared to that of Asian women, the milk of Caucasians had significantly lower levels of lactate (*p* = 0.03) and fucose (*p* = 0.03).

## 4. Discussion

In this comparison of 109 human milk samples from five countries, clustering by country of origin was observed, such that milk metabolites in South African women differed substantially from those in Norwegian and US women, and women in either of the two Australian cohorts. Milk metabolites in Japanese women formed an overlapping cluster between Norway and South Africa. Creatine and 2-oxoglutarate were the milk metabolites mainly responsible for these regional differences; they were also the highest in South African women among the cohort. Differences in these milk metabolites were evident in a comparison of healthy, non-atopic women in all countries studied; lactose milk levels were also the highest in South African women. Since several low molecular weight metabolites levels are tightly controlled in human milk [3], identifying differences between maternal countries of origin are noteworthy. In the pursuant paragraphs, we summarize what is known about these metabolites, highlight other findings, and offer candidate explanations for the higher levels seen in South African women when compared to women living in Australia or the northern hemisphere. 

After protein, lactose is the most plentiful component of human milk and often measured to reflect the carbohydrate energy content. Human milk lactose is found to be associated with infant growth in observational and simulation studies [22,23]. Since levels rise during the postpartum period in breastfeeding women [24], lactose has also been labelled as a marker for milk production. In our comparison, all human milk samples were obtained within one month of birth, removing timing of collection as an explanation for differences in milk lactose or other metabolite concentrations. In two to five months old infants, Gridneva et al. documented higher lactose milk levels in Australian women who breastfed more frequently [16]. Their results are congruent with a metabolomics study of sow milk, whereby Tan et al. reported higher milk lactose levels with higher rather than lower lactation performance [25]. Glucose and fucose, other sugars typically found in human milk, did not differ in their levels across country cohorts in our study. Lactose has also been found to have antimicrobial and innate immunity-inducing properties [2,26,27]. Hence, the much greater levels of milk lactose in South African women than in women living in more industrialized societies may reflect a greater breastfeeding frequency, or maternal programming to prevent infection.

Although milk sugars are the main substrates for energy generation and the production of oligosaccharides [28], little is known about these intermediate metabolites of the tricarboxylic acid energy cycle (TCA cycle), such as 2-oxoglutarate, citrate, or succinate. In our study, lactose levels were positively correlated with milk citrate and 2-oxoglutarate. Milk 2-oxoglutarate and citrate levels were also highest in South African women in our comparison; however, no differences were observed in milk succinate concentrations. As shown in a study of US women, the levels of milk 2-oxoglutarate in women feeding term infants typically decline within one month of birth [20]. Of note, lower 2-oxoglutarate levels have been detected in the milk of dairy cows with mastitis (breast duct infection), which is interpreted to be a function of greater consumption of this TCA cycle intermediate by resident microbiota or infecting microbes [3,29]. Mastitis and subclinical mastitis are common infections postpartum in industrialized countries [30], which offers another explanation for 2-oxoglutarate consumption and lower milk concentrations in US, Australian, and Norwegian women. Japanese women had higher milk levels of other energy-related intermediates such as lactate and pyruvate than Norwegian women; not much is known about the milk levels of these metabolites in humans. Compared to non-lactating cows, the TCA and related cycles, including pyruvate metabolism, were found to be most activated in the mammary glands of lactating cows [31]; pyruvate and lactate were among the 118 metabolites shared between the mammary gland and milk.

Lactose is produced by mammary cells, but other low-weight molecules are speculated to diffuse into human milk and serve as indicators of maternal plasma levels [2]. Methanol is found in human milk and can originate from maternal circulation following the consumption of fruits, vegetables, alcohol, and artificial sweeteners, and from exposure to environmental tobacco smoke [32]. Other examples are betaine, a choline metabolite whose levels increase with dietary intake of choline [33]. Consistently, milk betaine and the phosphocholines were positively correlated in our study. We also found milk methanol to be lowest in Norwegian women, whereas betaine levels were lowered in Australian milk. Milk creatinine (derived from creatine, an energy metabolite) levels are also related to circulating blood levels [34]; urinary creatinine also reportedly rises postpartum during breastfeeding [24]. In lactating cows, creatine and lactose are the two metabolites found in the stomach, serum, milk, and urine, denoting their importance in the lactation process [31]. Of note, betaine is an osmolytic and its supplementation can increase milk yield in cows [35]. Together with the higher levels of milk lactose and betaine, elevated levels of milk creatine or its metabolite, creatinine, may indicate higher milk production in South African women. 

In Tan et al.’s study of sow milk [25], glutamate and glutamine were additional markers of high milk production. Glutamate is produced when 2-oxoglutarate combines with glutamine. Glutamine and glutamate are the most abundant amino acids in human milk, increasing with each stage of lactation, and are largely derived during lactation from muscle protein breakdown of glutamine [21,36]. Higher levels of the amino acid valine have also been found to be associated with higher bovine milk yield [37]. It is then noteworthy that glutamate was the metabolite which distinguished US human milk composition from that of Norwegian and Japanese women. Milk glutamine levels were significantly lower in Norwegian and Japanese than in US women, which is consistent with the general observation that glutamine levels are higher in North America than in Asia [21]. Both glutamine and glutamate are important energy sources for intestinal cells and are needed for infant growth. When Dangat et al. compared healthy women in India to women with maternal conditions such as pre-eclampsia, which are associated with growth retardation in offspring, they found healthy women to have higher glutamine and glutamate levels in their milk, as well as higher lactose concentrations [38]. 

Ethnic differences between the same milk metabolome of mixed Black and Caucasian women were consistent with the country variation we observed, and further indicated a similarity among Caucasian women in Norway, Australia, and the US, potentially in terms of breastfeeding practices and/or maternal diet, health status, or genetics. Maternal diet has a reported influence on some human milk constituents, such as fatty acids, vitamins, and minerals; maternal intake of carbohydrates or fat is unrelated to milk lactose levels, but little is known about other low molecular weight substrates [13]. Since several low molecular metabolites detected in milk can be consumed or produced by resident microbiota, ethnic differences in these milk metabolites may also be a function of variation in human milk microbiota. As shown by Kumar et al., human milk microbial composition can vary according to the degree of societal industrialization, whereby the abundance of Proteobacteria is observed to be higher in milk samples of South African women [4]. In this regard, human milk lactose levels have been reported by others to vary inversely with milk Enterobacteria [26]. Further, maternal intake of lactose has been inversely correlated with milk concentrations within the Firmicutes phylum [39]. Unfortunately, data on maternal diet was not collected at the time that human milk samples were obtained in our study. Additionally, no women of Asian ethnicity were present in the Australian or US cohorts, and too few non-atopic women of Black race were recruited in the US cohort to speculate on genetic versus environmental origins of the milk metabolite variation.

The strengths of this international comparative study are that all human milk samples were selected at the same stage of lactation with similar maternal characteristics, and processed with NMR spectroscopy in one center. As has been reported for other milk constituents [40], despite not following an identical protocol for milk collection, the individual country samples were similar across several milk metabolites whose levels have been reported to be tightly controlled [20].

## 5. Conclusions

Human milk composition is optimal for infant growth and development. As we and others seek to determine how societal factors impact on the infant gut microbiome, immune development and subsequent health, understanding population differences in early nutrition is essential. This present work should be considered a first step in helping to frame breastfeeding/microbiome studies in a global context. It reminds us that regional maternal diet and breastfeeding practices have the capacity to influence milk composition. In addition to trying to understand this variation, we must also leverage study findings to inform policy, especially in times of change. In our study, the milk composition of South African women had higher levels of lactose, creatine and energy metabolites. How will South African infants fare against the rising trends of not being fed colostrum, receiving short exclusive breastfeeding and being introduced to solids at an early stage [41] in a country historically known for lower infant growth failure rates than it neighbors [42]? Understanding people and their place and purpose on this planet is essential to understanding the complexity we face in harnessing the health of the microbiome.

## Figures and Tables

**Table 1:** Comparison of milk metabolites concentrations (µmol/L) by country of origin.

MEAN (SD)					
Metabolites/Countries	South Africa	Japan	Norway	US	Perth #2
2-Oxoglutarate	79.6 (25.6)	79.1 (49.2) ^##^	30.5 (31.2) ***	39.0 (12.7) *	47.5 (19.9)
Acetone	13.2 (6.4)	19.8 (15.7)	10.8 (7.6)	11.4 (3.3)	13.3 (7.5)
Alanine	161.0 (93.5)	233.6 (96.8)	208.1 (84.5)	240.1 (91.9)	203.2 (48.4)
Betaine	668.1 (201.8)	355.3 (367.1)	180.4 (318.8) ***	408.4 (567.8)	58.8 (6.6) ***
Caprate	108.6 (124.7)	193.1 (216.3)	121.5 (120.3)	60.0 (90.6)	114.0 (42.9)
Caprylate	157.1 (137.2)	357.6 (497.0)	131.6 (207.1)	92.7 (132.5)	82.7 (47.1)
Choline	322.1 (194.9)	192.0 (128.3)	184.1 (163.7)	133.0 (77.0)	138.5 (71.1)
Citrate	5614 (3920)	2830 (1313) *	2718 (1361) ***	3642 (1272)	2856 (836.1) *
Creatine	274.7 (342.9)	60.3 (28.4) *	63.9 (26.9) ***	56.8 (23.4) *	66.5 (19.0) *
Creatine phosphate	49.6 (54.3)	45.2 (31.1)	20.6 (13.1) *	42.0 (12.7)	23.9 (7.6)
Creatinine	108.8 (82.7)	42.9 (15.4) **	43.7 (15.6) ***	57.3 (22.9) *	45.0 (5.7) **
Formate	83.4 (79.4)	112.2 (127.8)	902.0 (1695.2)	91.9 (55.7)	127.1 (24.9)
Fucose	278.5 (373.5)	570.9 (482.9)	330.5 (252.5)	344.4 (216.4)	382.3 (277.5)
Glucose	1347 (852.9)	1563 (1327)	926.4 (747.8)	1697 (413.5)	1699 (935.9)
Glutamate	858.9 (491.3)	1296 (565.8)	1467 (835.2)	1573 (830.2)	1554 (369.4)
Glutamine	282.0 (150.9)	103.0 (88.1)	101.9 (119.0)	514.2 (618.2) ^###^	207.6 (178.2)
Glycine	2796 (759.9)	3135 (1373)	2512 (1191)	1641 (1777)	1919 (949.5)
Guanidoacetate	4771 (1048)	3777 (1747)	3373 (2944)	3435 (1770)	1622 (286.5) *
Isoleucine	20.8 (12.2)	24.1 (25.9)	24.2 (21.1)	10.9 (2.6)	12.0 (4.0)
Lactate	213.7 (63.1)	3215 (5561) **^##^	443.5 (693.6)	137.8 (102.0)	125.7 (41.5)
Lactose	189,874 (34,234)	139,161 (83,333)	101,351 (84,404) *	161,160 (123,638)	83,524 (23,509) *
Leucine	33.7 (21.1)	49.9 (61.2)	66.2 (76.2)	30.9 (8.9)	34.0 (7.8)
Methanol	78.0 (17.6)	97.7 (49.2) ^###^	46.1 (19.3) ***	89.2 (6.7) ^###^	72.0 (6.8) ^#^
Phosphocholine	636.9 (328.6)	784.7 (470.6)	463.7 (326.7)	698.3 (231.2)	488.4 (145.6)
Pyruvate	53.3 (29.5)	68.6 (69.8) ^##^	20.0 (26.3) *	31.7 (39.7)	6.3 (2.5) *
Succinate	48.3 (67.0)	47.9 (25.9)	210.8 (515.7)	21.2 (9.6)	49.3 (15.7)
Valine	35.3 (11.9)	63.9 (33.5)	65.6 (32.7) *	50.2 (21.1)	57.4 (17.6)
Glycerophosphocholine	797.6 (439.3)	645.0 (393.0)	414.9 (346.0) *	589.2 (239.7)	517.4 (118.5)
# metabolite differences with South Africa	na	4	11	3	7
# metabolite differences with Norway	-	4	na	2	1

* *p* < 0.05, ** *p* < 0.01, *** *p* < 0.001 when reference = South Africa; ^#^ *p* < 0.05, ^##^ *p* < 0.01, ^###^ *p* < 0.001 when reference = Norway; na: not applicable.

**Table 2:** Comparison of milk metabolite concentrations (µmol/L) by maternal ethnicity.

MEAN (SD)			
Metabolites/Countries	Black	Caucasian	Asian
2-Oxoglutarate	75.4 (31.1)	37.6 (29.2) **	55.3 (47.1)
Acetone	12.9 (5.3)	11.9 (7.4)	14.2 (13.0)
Alanine	164.4 (87.9)	210.6 (82.1)	222.3 (90.9)
Betaine	581.1 (283.6)	204.9 (352.7) *	354.3 (390.1)
Caprate	77.2 (87.8)	109.8 (112.3)	185.1 (168.3)
Caprylate	134.7 (136.2)	118.1 (184.1)	266.9 (375.3)
Choline	285.1 (167.0)	170.2 (154.4)	218.7 (145.5)
Citrate	4892 (3687)	3123 (1833)	2776 (1061)
Creatine	229.4 (326.5)	77.6 (101.1) *	63.2 (28.5) *
Creatine phosphate	54.0 (56.2)	24.1 (13.7) **	35.9 (26.8)
Creatinine	89.7 (68.2)	51.6 (37.0) *	44.2 (15.0) *
Formate	86.5 (79.3)	582.6 (1363)	534.5 (1350)
Fucose	328.0 (376.9)	306.0 (236.6) ^#^	556.4 (391.5)
Glucose	1264 (672.2)	1263 (866.0)	1148 (1083)
Glutamate	1021 (614.5)	1478 (791.6)	1272 (536.3)
Glutamine	252.4 (122.0)	204.7 (310.6)	89.5 (79.2)
Glycine	2646 (1051)	2360 (1307)	2596 (1169)
Guanidoacetate	4405 (1378)	2912 (2538)	4544 (1916)
Isoleucine	17.3 (11.5)	20.5 (18.4)	23.4 (21.8)
Lactate	198.2 (82.2)	339.5 (600.5) ^#^	1918 (4213)
Lactose	178,365 (50,934)	110,060 (86,787)	131,284 (85,509)
Leucine	31.5 (14.8)	52.1 (56.4)	64.0 (87.0)
Methanol	80.4 (15.7)	59.3 (23.6)	71.0 (46.6)
Phosphocholine	584.6 (305.1)	521.9 (304.1)	642.5 (435.6)
Pyruvate	46.5 (25.3)	21.4 (29.6)	47.2 (58.8)
Succinate	36.1 (53.0)	152.7 (446.7)	100.8 (124.4)
Valine	34.6 (12.0)	61.1 (26.2) *	63.9 (40.5)
Glycerophosphocholine	846.4 (424.6)	467.7 (319.9) **	518.9 (327.7)
# metabolite differences with Black race	na	7	2
# metabolite differences with Asian race	3	na	2

* *p* < 0.05, ** *p* < 0.01, when reference = Black; ^#^ *p* < 0.05, when reference = Asian; na: not applicable.

## References

[1] Hennet T., Borsig L. (2016). Breastfed at tiffany’s. Trends Biochem. Sci..

[2] Demmelmair H., Koletzko B. (2017). Variation of metabolite and hormone contents in human milk. Clin. Perinatol..

[3] Smilowitz J.T., O’Sullivan A., Barile D., German J.B., Lonnerdal B., Slupsky C.M. (2013). The human milk metabolome reveals diverse oligosaccharide profiles. J. Nutr..

[4] Kumar H., du Toit E., Kulkarni A., Aakko J., Linderborg K.M., Zhang Y., Nicol M.P., Isolauri E., Yang B., Collado M.C. (2016). Distinct patterns in human milk microbiota and fatty acid profiles across specific geographic locations. Front. Microbiol..

[5] Fu Y., Liu X., Zhou B., Jiang A.C., Chai L. (2016). An updated review of worldwide levels of docosahexaenoic and arachidonic acid in human breast milk by region. Public Health Nutr..

[6] Victora C.G., Adair L., Fall C., Hallal P.C., Martorell R., Richter L., Sachdev H.S. (2008). Maternal and child undernutrition: Consequences for adult health and human capital. Lancet.

[7] Adair L.S., Fall C.H., Osmond C., Stein A.D., Martorell R., Ramirez-Zea M., Sachdev H.S., Dahly D.L., Bas I., Norris S.A. (2013). Associations of linear growth and relative weight gain during early life with adult health and human capital in countries of low and middle income: Findings from five birth cohort studies. Lancet.

[8] Garza C., Borghi E., Onyango A.W., de Onis M. (2013). Parental height and child growth from birth to 2 years in the WHO multicentre growth reference study. Matern. Child Nutr..

[9] Victora C.G., Bahl R., Barros A.J., Franca G.V., Horton S., Krasevec J., Murch S., Sankar M.J., Walker N., Rollins N.C. (2016). Breastfeeding in the 21st century: Epidemiology, mechanisms, and lifelong effect. Lancet.

[10] Munblit D., Peroni D.G., Boix-Amoros A., Hsu P.S., Van’t Land B., Gay M.C.L., Kolotilina A., Skevaki C., Boyle R.J., Collado M.C. (2017). Human milk and allergic diseases: An unsolved puzzle. Nutrients.

[11] Davis J.C., Totten S.M., Huang J.O., Nagshbandi S., Kirmiz N., Garrido D.A., Lewis Z.T., Wu L.D., Smilowitz J.T., German J.B. (2016). Identification of oligosaccharides in feces of breast-fed infants and their correlation with the gut microbial community. Mol. Cell Proteomics.

[12] Koleva P.T., Bridgman S.L., Kozyrskyj A.L. (2015). The infant gut microbiome: Evidence for obesity risk and dietary intervention. Nutrients.

[13] Bravi F., Wiens F., Decarli A., Dal Pont A., Agostoni C., Ferraroni M. (2016). Impact of maternal nutrition on breast-milk composition: A systematic review. Am. J. Clin. Nutr..

[14] Wahlqvist M.L. (2015). Lactose nutrition in lactase nonpersisters. Asia Pac. J. Clin. Nutr..

[15] Taylor A.L., Dunstan J.A., Prescott S.L. (2007). Probiotic supplementation for the first 6 months of life fails to reduce the risk of atopic dermatitis and increases the risk of allergen sensitization in high-risk children: A randomized controlled trial. J. Allergy Clin. Immunol..

[16] Gridneva Z., Kugananthan S., Hepworth A.R., Tie W.J., Lai C.T., Ward L.C., Hartmann P.E., Geddes D.T. (2016). Effect of human milk appetite hormones, macronutrients, and infant characteristics on gastric emptying and breastfeeding patterns of term fully breastfed infants. Nutrients.

[17] Wegienka G., Havstad S., Joseph C.L., Zoratti E., Ownby D., Woodcroft K., Johnson C.C. (2012). Racial disparities in allergic outcomes in african americans emerge as early as age 2 years. Clin. Exp. Allergy.

[18] Eggesbo M., Thomsen C., Jorgensen J.V., Becher G., Odland J.O., Longnecker M.P. (2011). Associations between brominated flame retardants in human milk and thyroid-stimulating hormone (TSH) in neonates. Environ. Res..

[19] Package “Corrplot”. https://cran.r-project.org/web/packages/corrplot/corrplot.pdf..

[20] Spevacek A.R., Smilowitz J.T., Chin E.L., Underwood M.A., German J.B., Slupsky C.M. (2015). Infant maturity at birth reveals minor differences in the maternal milk metabolome in the first month of lactation. J. Nutr..

[21] Zhang Z., Adelman A.S., Rai D., Boettcher J., Lonnerdal B. (2013). Amino acid profiles in term and preterm human milk through lactation: A systematic review. Nutrients.

[22] Prentice P., Ong K.K., Schoemaker M.H., van Tol E.A., Vervoort J., Hughes I.A., Acerini C.L., Dunger D.B. (2016). Breast milk nutrient content and infancy growth. Acta Paediatr..

[23] Nilsson A., Mardinoglu A., Nielsen J. (2017). Predicting growth of the healthy infant using a genome scale metabolic model. NPJ Syst. Biol. Appl..

[24] Sachse D., Baerug A., Sletner L., Birkeland K.I., Nakstad B., Jenum A.K., Berg J.P. (2014). Urine nmr metabolomics analysis of breastfeeding biomarkers during and after pregnancy in a large prospective cohort study. Scand. J. Clin. Lab. Investig..

[25] Tan C., Zhai Z., Ni X., Wang H., Ji Y., Tang T., Ren W., Long H., Deng B., Deng J. (2018). Metabolomic profiles reveal potential factors that correlate with lactation performance in sow milk. Sci. Rep..

[26] Boix-Amoros A., Collado M.C., Mira A. (2016). Relationship between milk microbiota, bacterial load, macronutrients, and human cells during lactation. Front. Microbiol..

[27] Cederlund A., Kai-Larsen Y., Printz G., Yoshio H., Alvelius G., Lagercrantz H., Stromberg R., Jornvall H., Gudmundsson G.H., Agerberth B. (2013). Lactose in human breast milk an inducer of innate immunity with implications for a role in intestinal homeostasis. PLoS ONE.

[28] Cocinero E.J., Carcabal P. (2015). Carbohydrates. Top. Curr. Chem..

[29] Xi X., Kwok L.Y., Wang Y., Ma C., Mi Z., Zhang H. (2017). Ultra-performance liquid chromatography-quadrupole-time of flight mass spectrometry ms(e)-based untargeted milk metabolomics in dairy cows with subclinical or clinical mastitis. J. Dairy Sci..

[30] Axelsson D., Blomberg M. (2014). Prevalence of postpartum infections: A population-based observational study. Acta. Obstet. Gynecol. Scand..

[31] Sun H.Z., Shi K., Wu X.H., Xue M.Y., Wei Z.H., Liu J.X., Liu H.Y. (2017). Lactation-related metabolic mechanism investigated based on mammary gland metabolomics and 4 biofluids’ metabolomics relationships in dairy cows. BMC Genom..

[32] Dorokhov Y.L., Shindyapina A.V., Sheshukova E.V., Komarova T.V. (2015). Metabolic methanol: Molecular pathways and physiological roles. Physiol. Rev..

[33] Fischer L.M., da Costa K.A., Galanko J., Sha W., Stephenson B., Vick J., Zeisel S.H. (2010). Choline intake and genetic polymorphisms influence choline metabolite concentrations in human breast milk and plasma. Am. J. Clin. Nutr..

[34] Balzer M.S., Gross M.M., Lichtinghagen R., Haller H., Schmitt R. (2015). Got milk? Breastfeeding and milk analysis of a mother on chronic hemodialysis. PLoS ONE.

[35] Peterson S.E., Rezamand P., Williams J.E., Price W., Chahine M., McGuire M.A. (2012). Effects of dietary betaine on milk yield and milk composition of mid-lactation holstein dairy cows. J. Dairy Sci..

[36] Manso H.E., Filho H.C., de Carvalho L.E., Kutschenko M., Nogueira E.T., Watford M. (2012). Glutamine and glutamate supplementation raise milk glutamine concentrations in lactating gilts. J. Anim. Sci. Biotechnol..

[37] Wu X., Sun H., Xue M., Wang D., Guan L.L., Liu J. (2018). Serum metabolome profiling revealed potential biomarkers for milk protein yield in dairy cows. J. Proteomics.

[38] Dangat K., Upadhyay D., Kilari A., Sharma U., Kemse N., Mehendale S., Lalwani S., Wagh G., Joshi S., Jagannathan N.R. (2016). Altered breast milk components in preeclampsia; an in-vitro proton nmr spectroscopy study. Clin. Chim. Acta.

[39] Williams J.E., Carrothers J.M., Lackey K.A., Beatty N.F., York M.A., Brooker S.L., Shafii B., Price W.J., Settles M.L., McGuire M.A. (2017). Human milk microbial community structure is relatively stable and related to variations in macronutrient and micronutrient intakes in healthy lactating women. J. Nutr..

[40] Munblit D., Treneva M., Peroni D.G., Colicino S., Chow L., Dissanayeke S., Abrol P., Sheth S., Pampura A., Boner A.L. (2016). Colostrum and mature human milk of women from London, Moscow, and Verona: Determinants of immune composition. Nutrients.

[41] Budree S., Goddard E., Brittain K., Cader S., Myer L., Zar H.J. (2017). Infant feeding practices in a south African birth cohort-a longitudinal study. Matern. Child Nutr..

[42] Osgood-Zimmerman A., Millear A.I., Stubbs R.W., Shields C., Pickering B.V., Earl L., Graetz N., Kinyoki D.K., Ray S.E., Bhatt S. (2018). Mapping child growth failure in Africa between 2000 and 2015. Nature.

